# (Benzoyl­acetonato-κ^2^ *O*,*O*′)dicarbonyl­rhodium(I)

**DOI:** 10.1107/S1600536812044893

**Published:** 2012-11-03

**Authors:** Carla Pretorius, Andreas Roodt

**Affiliations:** a Department of Chemistry, University of the Free State, PO Box 339, Bloemfontein 9300, South Africa

## Abstract

In the title compound, [Rh(C_10_H_9_O_2_)(CO)_2_], a distorted square-planar coordination geometry is observed around the Rh^I^ atom, formed by the O atoms of the bidentate ligand and two C atoms from the carbonyl ligands. The Rh^I^ atom is displaced from the plane through the surrounding atoms by 0.017 Å. In the crystal, C—H⋯O inter­action is observed between a methyl group of the bidentate ligand and a carbonyl O atom. Metallophilic inter­actions [3.308 (3) and 3.461 (3) Å] between neighbouring Rh^I^ atoms are encountered in the crystal, resulting in the formation of a metal chain along the *b-*axis direction.

## Related literature

For applications of rhodium chemistry, see: Dutta & Singh (1994 ▶); Paulik & Roth (1968 ▶); Evans *et al.* (1968 ▶). For rhodium dicarbonyl complexes as precursor catalysts, see: Brink *et al.* (2010 ▶). For background to metallophilicity, see: Doerrer (2010 ▶). For other metallophilic rhodium complexes, see: Prater *et al.* (1999 ▶); Laurila *et al.* (2012 ▶); Real *et al.* (1989 ▶). For other rhodium dicarbonyl complexes, see: Huq & Skapski (1974 ▶); Leipoldt *et al.* (1977 ▶).
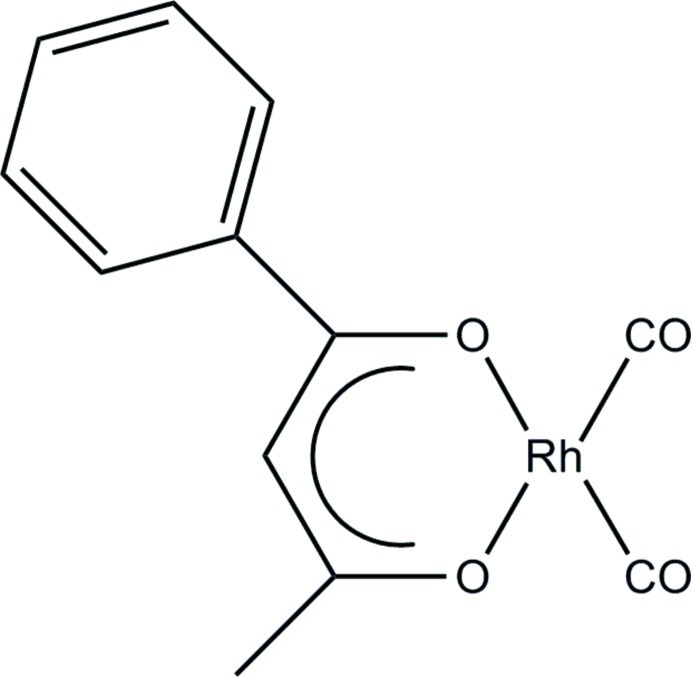


## Experimental

### 

#### Crystal data

[Rh(C_10_H_9_O_2_)(CO)_2_]
*M* *_r_* = 320.1
Monoclinic, 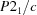

*a* = 7.5887 (2) Å
*b* = 6.7522 (1) Å
*c* = 22.5299 (5) Å
β = 98.850 (1)°
*V* = 1140.70 (4) Å^3^
*Z* = 4
Mo *K*α radiation
μ = 1.50 mm^−1^
*T* = 100 K
0.19 × 0.09 × 0.05 mm

#### Data collection

Bruker APEXII KappaCCD diffractometer
Absorption correction: multi-scan (*SADABS*; Bruker, 2008 ▶) *T*_min_ = 0.851, *T*_max_ = 0.928
15332 measured reflections
2827 independent reflections
2494 reflections with *I* > 2σ(*I*)
*R*_int_ = 0.020

#### Refinement

*R*[*F* ^2^ > 2σ(*F* ^2^)] = 0.018
*wR*(*F* ^2^) = 0.048
*S* = 1.08
2827 reflections
154 parameters
H-atom parameters constrained
Δρ_max_ = 0.56 e Å^−3^
Δρ_min_ = −0.44 e Å^−3^

### 

Data collection: *APEX2* (Bruker, 2011 ▶); cell refinement: *SAINT-Plus* (Bruker, 2008 ▶); data reduction: *SAINT-Plus*; program(s) used to solve structure: *SIR2002* (Burla *et al.*, 2003 ▶) and *SHELXS97* (Sheldrick, 2008 ▶); program(s) used to refine structure: *SHELXL97* (Sheldrick, 2008 ▶); molecular graphics: *Mercury* (Macrae *et al.*, 2008 ▶); software used to prepare material for publication: *WinGX* (Farrugia, 1999 ▶), *publCIF* (Westrip, 2010 ▶), *PARST* (Nardelli, 1995 ▶) and *PLATON* (Spek, 2009 ▶).

## Supplementary Material

Crystal structure: contains datablock(s) global, I. DOI: 10.1107/S1600536812044893/bh2458sup1.cif

Structure factors: contains datablock(s) I. DOI: 10.1107/S1600536812044893/bh2458Isup2.hkl

Additional supplementary materials:  crystallographic information; 3D view; checkCIF report

## Figures and Tables

**Table 1:** Hydrogen-bond geometry (Å, °)

*D*—H⋯*A*	*D*—H	H⋯*A*	*D*⋯*A*	*D*—H⋯*A*
C10—H10*A*⋯O3^i^	0.98	2.57	3.427 (2)	146

Symmetry code: (i) 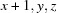.

**Fig. 1.**
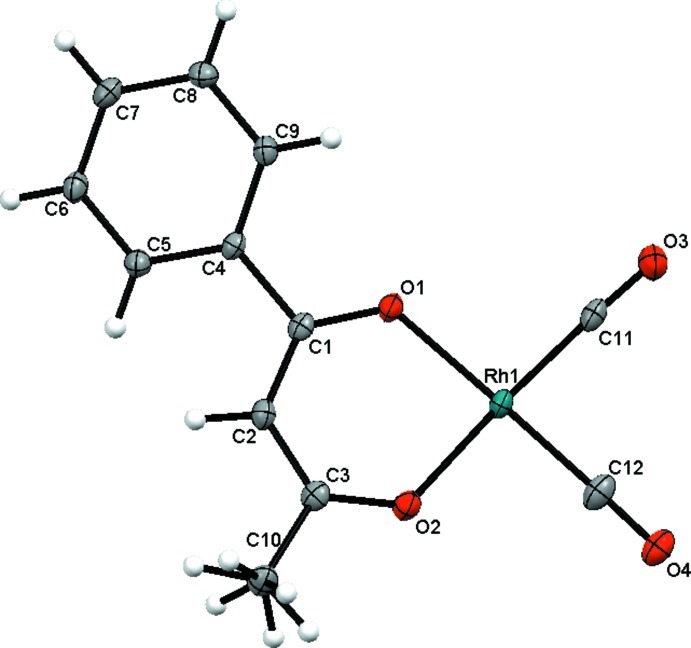
Molecular structure of the title compound. Displacement ellipsoids are drawn at the 50% probability level.

**Fig. 2.**
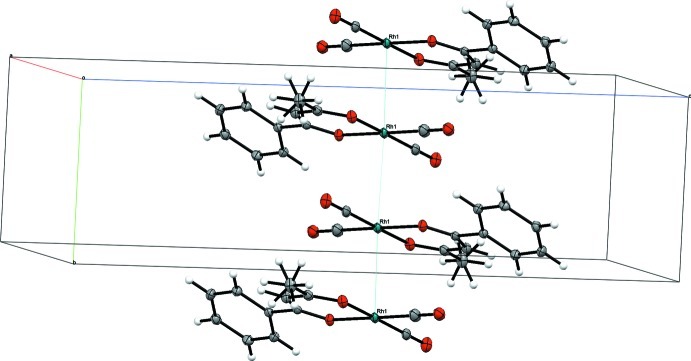
The metallophilic Rh···Rh interactions result in the formation of a 1D metal chain along the b-axis.

## supplementary crystallographic information

### Comment

Rhodium is one of the most studied transition metals due to its importance in various applications including catalysis and biological activity (Dutta & Singh, 1994). It is widely recognized as a good catalyst for several industrial processes such as the Monsanto process (Paulik & Roth, 1968) and hydroformylation (Evans *et al.*, 1968). In turn, rhodium dicarbonyl complexes of the type [Rh(*L*,*L*')(CO)_2_] where (*L*,*L*') represents a mono-anionic bidentate ligand have been widely studied as catalyst precursors (Brink *et al.*, 2010).

The structural analysis of the title complex was undertaken to obtain a better understanding of the rhodium-ligand interactions in this type of compound and as a partial study of the effects that the different substituents in non-symmetrical *β*-diketones have on the kinetics of substitution reactions of these complexes.

Metallophilicity has been defined as the interaction between electron densities of large metal centres with an associated energy in the same order as hydrogen-bonding (Doerrer, 2010). These metallophilic interactions lead to the construction of 1D metal chains and have been widely recognized for other square-planar Rh^I^ molecules (Prater *et al.*, 1999; Laurila *et al.*, 2012; Real *et al.*, 1989). The rhodium complex reported here showed stacking in such a way that the rhodium atoms of neighbouring molecules occupy the two remaining pseudo-octahedral positions almost perpendicular to the coordination polyhedron, with Rh···Rh distances of 3.308 (3) and 3.461 (3) Å respectively (see Figure 2). These values are slightly longer than the Rh···Rh distances reported for [Rh(acac)(CO)_2_] (3.253 and 3.271 Å, Huq & Skapski, 1974). For the benzoyl-1,1,1-trifluoroacetonatodicarbonylrhodium(I) complex these distances were reported as 3.537 Å (Leipoldt *et al.*, 1977). The metallophilic interactions result in the formation of a 1D metal chain along the *b*-axis in the unit cell. This is consistent with the [Rh(acac)(CO)_2_] complex and benzoyl-1,1,1-trifluoroacetonatodicarbonylrhodium(I), that also displayed chain growth along the shortest unit cell axis.

A substitutional disorder over two positions was observed for the hydrogen atoms of the methyl group on the pentenone backbone. Intermolecular C10—H10C···O3 hydrogen bonding in the order of 3.427 Å was observed with one of the carbonyl moieties.

### Experimental

[RhCl(CO)_2_]_2_ was prepared *in situ* by refluxing RhCl_3_.3H_2_O (0.1014 g, 0.385 mmol) in 2 ml DMF for 30 min. 1-Phenyl-1,3-butanedione (0.0906 g, 0.905 mmol) was added to the cooled DMF solution of [RhCl(CO)_2_]_2_. The orange product was precipitated with ice-water and isolated by centrifuge. Recrystallization from diethyl ether yielded pleochroic orange-red crystals suitable for X-ray diffraction. IR (ATR, cm^-1^): *ν_CO, sym_* 2066 s, *ν_CO, asym_* 1999 s.

### Refinement

The methyl and aromatic H atoms were placed in geometrically idealized positions and constrained to ride on their parent atoms, with C—H = 0.95 and 0.98 Å and *U*_iso_(H) = 1.5*U*_eq_(carrier C) and 1.2*U*_eq_(carrier C), respectively. The highest residual electron density was located 0.04 Å from C12 and the deepest hole was 0.17 Å from O4.

### Crystal data

**Table:**

[Rh(C_10_H_9_O_2_)(CO)_2_]	*F*(000) = 632
*M**_r_* = 320.1	*D*_x_ = 1.864 Mg m^−^^3^
Monoclinic, *P*2_1_/*c*	Mo *K*α radiation, λ = 0.71073 Å
Hall symbol: -P 2ybc	Cell parameters from 7402 reflections
*a* = 7.5887 (2) Å	θ = 2.7–28.3°
*b* = 6.7522 (1) Å	µ = 1.50 mm^−^^1^
*c* = 22.5299 (5) Å	*T* = 100 K
β = 98.850 (1)°	Needle, orange
*V* = 1140.70 (4) Å^3^	0.19 × 0.09 × 0.05 mm
*Z* = 4	

### Data collection

**Table:**

Bruker APEXII KappaCCD diffractometer	2827 independent reflections
Radiation source: fine-focus sealed tube	2494 reflections with *I* > 2σ(*I*)
Graphite monochromator	*R*_int_ = 0.020
Detector resolution: 512 pixels mm^-1^	θ_max_ = 28.3°, θ_min_ = 1.8°
φ and ω scans	*h* = −10→10
Absorption correction: multi-scan (*SADABS*; Bruker, 2008)	*k* = −9→8
*T*_min_ = 0.851, *T*_max_ = 0.928	*l* = −29→30
15332 measured reflections	

### Refinement

**Table:**

Refinement on *F*^2^	Primary atom site location: structure-invariant direct methods
Least-squares matrix: full	Secondary atom site location: difference Fourier map
*R*[*F*^2^ > 2σ(*F*^2^)] = 0.018	Hydrogen site location: inferred from neighbouring sites
*wR*(*F*^2^) = 0.048	H-atom parameters constrained
*S* = 1.08	*w* = 1/[σ^2^(*F*_o_^2^) + (0.0197*P*)^2^ + 0.986*P*] where *P* = (*F*_o_^2^ + 2*F*_c_^2^)/3
2827 reflections	(Δ/σ)_max_ = 0.002
154 parameters	Δρ_max_ = 0.56 e Å^−^^3^
0 restraints	Δρ_min_ = −0.44 e Å^−^^3^
0 constraints	

### Fractional atomic coordinates and isotropic or equivalent isotropic displacement parameters (Å^2^)

**Table:**

	*x*	*y*	*z*	*U*_iso_*/*U*_eq_	Occ. (<1)
C1	−0.0248 (2)	0.2092 (2)	0.36980 (7)	0.0130 (3)	
C2	0.1566 (2)	0.1653 (2)	0.37257 (7)	0.0156 (3)	
H2	0.1964	0.1341	0.3357	0.019*	
C3	0.2848 (2)	0.1629 (2)	0.42418 (8)	0.0157 (3)	
C4	−0.1396 (2)	0.2156 (2)	0.30958 (7)	0.0132 (3)	
C5	−0.0945 (2)	0.1102 (2)	0.26078 (7)	0.0162 (3)	
H5	0.0096	0.0299	0.2658	0.019*	
C6	−0.2006 (2)	0.1218 (3)	0.20490 (8)	0.0174 (3)	
H6	−0.1681	0.0505	0.1719	0.021*	
C7	−0.3540 (2)	0.2371 (2)	0.19702 (8)	0.0171 (3)	
H7	−0.4262	0.2448	0.1587	0.02*	
C8	−0.4015 (2)	0.3411 (3)	0.24546 (8)	0.0170 (3)	
H8	−0.5067	0.4195	0.2403	0.02*	
C9	−0.2949 (2)	0.3305 (2)	0.30142 (7)	0.0150 (3)	
H9	−0.3278	0.4019	0.3344	0.018*	
C10	0.4773 (2)	0.1331 (3)	0.41774 (8)	0.0201 (4)	
H10A	0.5507	0.1351	0.4575	0.03*	0.5
H10B	0.5155	0.2396	0.393	0.03*	0.5
H10C	0.4912	0.0052	0.3984	0.03*	0.5
H10D	0.4876	0.1182	0.3751	0.03*	0.5
H10E	0.5227	0.0136	0.4396	0.03*	0.5
H10F	0.5471	0.2481	0.4342	0.03*	0.5
C11	−0.1975 (2)	0.3058 (3)	0.53050 (7)	0.0170 (3)	
C12	0.1228 (2)	0.2372 (2)	0.58225 (8)	0.0177 (3)	
O1	−0.10332 (17)	0.25034 (16)	0.41481 (5)	0.0152 (2)	
O2	0.25558 (16)	0.18797 (19)	0.47805 (5)	0.0170 (2)	
O3	−0.32389 (17)	0.3454 (2)	0.54935 (6)	0.0240 (3)	
O4	0.19329 (19)	0.23292 (19)	0.63064 (6)	0.0245 (3)	
Rh1	0.013973 (17)	0.244320 (18)	0.503043 (5)	0.01307 (5)	

### Atomic displacement parameters (Å^2^)

**Table:**

	*U*^11^	*U*^22^	*U*^33^	*U*^12^	*U*^13^	*U*^23^
C1	0.0169 (8)	0.0101 (7)	0.0118 (7)	−0.0026 (6)	0.0018 (6)	0.0010 (6)
C2	0.0181 (8)	0.0165 (8)	0.0127 (8)	−0.0007 (6)	0.0040 (6)	0.0006 (6)
C3	0.0172 (8)	0.0140 (8)	0.0160 (8)	−0.0023 (6)	0.0028 (6)	0.0020 (6)
C4	0.0157 (8)	0.0130 (7)	0.0108 (7)	−0.0020 (6)	0.0018 (6)	0.0010 (6)
C5	0.0181 (8)	0.0160 (8)	0.0146 (8)	0.0024 (6)	0.0026 (6)	0.0001 (6)
C6	0.0230 (9)	0.0169 (8)	0.0124 (8)	0.0005 (7)	0.0033 (7)	−0.0023 (6)
C7	0.0204 (8)	0.0172 (8)	0.0126 (8)	−0.0014 (6)	−0.0006 (6)	0.0018 (6)
C8	0.0169 (8)	0.0163 (8)	0.0174 (8)	0.0024 (6)	0.0014 (7)	0.0014 (6)
C9	0.0178 (8)	0.0141 (8)	0.0138 (8)	−0.0003 (6)	0.0044 (6)	−0.0012 (6)
C10	0.0148 (8)	0.0282 (9)	0.0170 (8)	−0.0028 (7)	0.0019 (7)	0.0007 (7)
C11	0.0239 (9)	0.0163 (8)	0.0100 (8)	−0.0033 (7)	0.0000 (7)	−0.0002 (6)
C12	0.0198 (8)	0.0142 (8)	0.0192 (9)	−0.0039 (6)	0.0038 (7)	−0.0002 (6)
O1	0.0166 (6)	0.0180 (6)	0.0111 (6)	−0.0011 (4)	0.0021 (5)	−0.0002 (4)
O2	0.0159 (6)	0.0216 (6)	0.0135 (6)	−0.0026 (5)	0.0020 (5)	0.0011 (5)
O3	0.0240 (7)	0.0308 (7)	0.0183 (6)	0.0009 (6)	0.0063 (5)	−0.0014 (6)
O4	0.0306 (8)	0.0264 (7)	0.0143 (6)	−0.0057 (5)	−0.0033 (6)	0.0003 (5)
Rh1	0.01542 (8)	0.01454 (7)	0.00917 (7)	−0.00290 (5)	0.00160 (5)	0.00006 (5)

### Geometric parameters (Å, º)

**Table:**

C1—O1	1.283 (2)	C8—C9	1.391 (2)
C1—C2	1.399 (2)	C8—H8	0.95
C1—C4	1.496 (2)	C9—H9	0.95
C2—C3	1.397 (2)	C10—H10A	0.98
C2—H2	0.95	C10—H10B	0.98
C3—O2	1.278 (2)	C10—H10C	0.98
C3—C10	1.504 (2)	C10—H10D	0.98
C4—C5	1.396 (2)	C10—H10E	0.98
C4—C9	1.400 (2)	C10—H10F	0.98
C5—C6	1.388 (2)	C11—O3	1.139 (2)
C5—H5	0.95	C11—Rh1	1.8539 (18)
C6—C7	1.389 (2)	C12—O4	1.138 (2)
C6—H6	0.95	C12—Rh1	1.8480 (19)
C7—C8	1.391 (2)	O1—Rh1	2.0498 (12)
C7—H7	0.95	O2—Rh1	2.0349 (12)
			
O1—C1—C2	125.71 (15)	H10A—C10—H10B	109.5
O1—C1—C4	115.70 (15)	C3—C10—H10C	109.5
C2—C1—C4	118.57 (15)	H10A—C10—H10C	109.5
C3—C2—C1	126.44 (15)	H10B—C10—H10C	109.5
C3—C2—H2	116.8	C3—C10—H10D	109.5
C1—C2—H2	116.8	H10A—C10—H10D	141.1
O2—C3—C2	126.05 (16)	H10B—C10—H10D	56.3
O2—C3—C10	114.98 (15)	H10C—C10—H10D	56.3
C2—C3—C10	118.95 (15)	C3—C10—H10E	109.5
C5—C4—C9	118.87 (15)	H10A—C10—H10E	56.3
C5—C4—C1	121.36 (15)	H10B—C10—H10E	141.1
C9—C4—C1	119.77 (15)	H10C—C10—H10E	56.3
C6—C5—C4	120.47 (16)	H10D—C10—H10E	109.5
C6—C5—H5	119.8	C3—C10—H10F	109.5
C4—C5—H5	119.8	H10A—C10—H10F	56.3
C5—C6—C7	120.36 (16)	H10B—C10—H10F	56.3
C5—C6—H6	119.8	H10C—C10—H10F	141.1
C7—C6—H6	119.8	H10D—C10—H10F	109.5
C6—C7—C8	119.72 (16)	H10E—C10—H10F	109.5
C6—C7—H7	120.1	O3—C11—Rh1	177.49 (15)
C8—C7—H7	120.1	O4—C12—Rh1	178.55 (17)
C9—C8—C7	120.02 (16)	C1—O1—Rh1	125.23 (11)
C9—C8—H8	120	C3—O2—Rh1	125.64 (11)
C7—C8—H8	120	C12—Rh1—C11	87.98 (8)
C8—C9—C4	120.54 (15)	C12—Rh1—O2	88.54 (7)
C8—C9—H9	119.7	C11—Rh1—O2	175.85 (6)
C4—C9—H9	119.7	C12—Rh1—O1	179.13 (6)
C3—C10—H10A	109.5	C11—Rh1—O1	92.88 (6)
C3—C10—H10B	109.5	O2—Rh1—O1	90.61 (5)

### Hydrogen-bond geometry (Å, º)

**Table:**

*D*—H···*A*	*D*—H	H···*A*	*D*···*A*	*D*—H···*A*
C10—H10*A*···O3^i^	0.98	2.57	3.427 (2)	146

Symmetry code: (i) *x*+1, *y*, *z*.

## References

[bb1] Brink, A., Roodt, A., Steyl, G. & Visser, H. G. (2010). *Dalton Trans.* **39**, 5572–5578. 10.1039/b922083f 20480082

[bb2] Bruker (2008). *SAINT-Plus* and *SADABS* Bruker AXS Inc., Madison, Wisconsin, USA.

[bb3] Bruker (2011). *APEX2* Bruker AXS Inc., Madison, Wisconsin, USA.

[bb4] Burla, M. C., Camalli, M., Carrozzini, B., Cascarano, G. L., Giacovazzo, C., Polidori, G. & Spagna, R. (2003). *J. Appl. Cryst.* **36**, 1103.

[bb5] Doerrer, L. H. (2010). *Dalton Trans.* **39**, 3543–3553. 10.1039/b920389c 20364462

[bb6] Dutta, D. K. & Singh, M. M. (1994). *Transition Met. Chem.* **19**, 290–292.

[bb7] Evans, D., Osborn, J. A. & Wilkinson, G. (1968). *J. Chem. Soc. A*, pp. 3133–3142.

[bb8] Farrugia, L. J. (1999). *J. Appl. Cryst.* **32**, 837–838.

[bb9] Huq, F. & Skapski, A. C. (1974). *J. Cryst. Mol. Struct.* **4**, 411–418.

[bb10] Laurila, E., Oresmaa, L., Hassinen, J., Hirva, P. & Haukka, M. (2012). *Dalton Trans.* In the press. doi:10.1039/C2DT31671D. 10.1039/c2dt31671d 23080011

[bb11] Leipoldt, J. G., Bok, L. D. C., Basson, S. S., van Vollenhoven, J. S. & Gerber, T. I. A. (1977). *Inorg. Chim. Acta*, **25**, L63–L64.

[bb12] Macrae, C. F., Bruno, I. J., Chisholm, J. A., Edgington, P. R., McCabe, P., Pidcock, E., Rodriguez-Monge, L., Taylor, R., van de Streek, J. & Wood, P. A. (2008). *J. Appl. Cryst.* **41**, 466–470.

[bb13] Nardelli, M. (1995). *J. Appl. Cryst.* **28**, 659.

[bb14] Paulik, F. E. & Roth, J. F. (1968). *Chem. Commun. (London)*, pp. 1578–1578.

[bb15] Prater, M. E., Pence, L. E., Clérac, R., Finniss, G. M., Campana, C., Auban-Senzier, P., Jérome, D., Canadell, E. & Dunbar, K. R. (1999). *J. Am. Chem. Soc.* **121**, 8005–8016.

[bb16] Real, J., Bayón, J. C., Lahoz, F. J. & López, J. A. (1989). *J. Chem. Soc. Chem. Commun.* pp. 1889–1890.

[bb17] Sheldrick, G. M. (2008). *Acta Cryst.* A**64**, 112–122. 10.1107/S0108767307043930 18156677

[bb18] Spek, A. L. (2009). *Acta Cryst.* D**65**, 148–155. 10.1107/S090744490804362X PMC2631630 19171970

[bb19] Westrip, S. P. (2010). *J. Appl. Cryst.* **43**, 920–925.

